# Advances in Human Neuroconnectivity Research: Applications for Understanding Familial History Risk for Alcoholism

**DOI:** 10.35946/arcr.v37.1.06

**Published:** 2015

**Authors:** Anita Cservenka, Gabriela Alarcón, Scott A. Jones, Bonnie J. Nagel

**Affiliations:** Anita Cservenka, Ph.D., is a postdoctoral fellow;; Gabriela Alarcón is a graduate student;; Scott A. Jones is a graduate student;; Bonnie J. Nagel, Ph.D., is associate professor of psychiatry and behavioral neuroscience, all at Oregon Health & Science University, Portland, Oregon.

**Keywords:** Alcoholism, genetic vulnerability, genetic risk factors, brain, brain function, brain imaging, neuroimaging, neuron, neural network, neuroconnectivity, neurobiology, functional magnetic resonance imaging, psychophysiological interactions, neuroscience

## Abstract

Recent advances in brain imaging have allowed researchers to further study the networks connecting brain regions. Specifically, research examining the functioning of these networks in groups with a genetic predisposition for alcoholism has found atypical circuitry in the brains of such individuals. Further research with larger sample sizes and multimodal method integration are necessary to confirm these intriguing findings.

Advances in human neuroimaging have expanded our ability to understand the functioning of the brain, with particular recent advances fostering our analytic capacity to examine networks between the brain’s nerve cells (i.e., neurons) and neuroconnectivity (i.e., neural networks). Relevant to the field of alcoholism, several researchers recently have applied these strategies to groups at genetic risk for alcoholism, in hopes of identifying neurobiological, and specifically neuroconnectivity, phenotypes underlying this risk. This article provides an overview of the methods used to study connectivity and highlights research detailing the application of these methods to studying populations at risk for alcoholism.

## Neuroconnectivity Methods

### Task-Based Connectivity

With the aim of understanding network functioning in the brain, one analytic strategy has been to examine the correlations between activation in regionally disparate brain regions during functional magnetic resonance imaging (fMRI).^1^ To do this, investigators typically have correlated average signal change in two or more regions of interest (ROIs) during a task, with the assumption that higher correlations reflect greater connectivity between regions (i.e., they are simultaneously showing significant changes in neural activation). Although this approach is confounded by variations in the underlying baseline intrinsic connectivity of the brain, it has nonetheless been used to demonstrate evidence of altered neuroconnectivity patterns in specific populations, as detailed below.

Psychophysiological interactions (PPI) analysis is another functional connectivity method used to analyze the coupling of neuronal activity between distinct brain regions while an individual is engaged in a task. This is different than other functional connectivity methods (e.g., resting-state functional connectivity) in that it allows one to assess the impact of task condition (or context) on the functional connectivity of two distinct brain regions. Friston and colleagues (1997) first described PPI as the statistical processing (i.e., regression) of neuronal activity in one brain region (the target region) onto the neuronal activity in a second (seed) region, with the slope of this regression being indicative of the relationship between the activity in these two regions. Comparing the slope of this regression during two distinct task conditions is the crux of PPI analysis. With PPI, seed regions can either be defined by functional subsets of data (i.e., masks) created in group-level analysis by selecting the volume elements (i.e., voxels)—three-dimensional elements that make up an image—that are most active during the task condition, or by a priori selection of a particular anatomical brain ROI (O’Reilly et al. 2012). The neural activity over time from this seed region is then multiplied by the regressor representing task-related activity and entered into the individual subject model to identify brain regions or voxels whose activity is synchronous with activity in the seed region. Recently, a more generalized form of PPI analysis allowed for the use of more than two task conditions in the same PPI model (McLaren et al. 2012). This is especially pertinent when analyzing tasks that have two or more distinct experimental conditions, as well as a control (baseline) condition in which no stimulus is occurring, typical of many fMRI tasks currently employed. This generalized form of PPI allows for better within-subject model fit and prevents having to collapse data across multiple conditions. Furthermore, PPI also can be used to compare functional connectivity between groups using group status, instead of context, as the regressor (O’Reilly et al. 2012) and could be useful for comparing groups with and without family history of alcoholism.

### Connectivity Without Task Engagement

The functional connectivity of the brain also has been measured using resting-state functional connectivity (RSFC) during fMRI. With this technique, functional connectivity is measured by correlating blood-oxygen-level–dependent (BOLD) signal, an indirect measure of neuronal activity, across the brain in an individual who is resting. Regions of the brain are thought to be functionally connected if they share a temporally correlated neurophysiologic response. Spontaneous brain fluctuations persist across a variety of states, such as sleep or anesthesia, as well as in animal species (Kannurpatti et al. 2008; Kojima et al. 2009), suggesting that spontaneous BOLD correlations are an intrinsic property of brain activity (Fox and Raichle 2007). RSFC has led to the identification of numerous sets of functionally connected brain regions, termed networks, including the default mode, fronto-parietal, dorsal attention, and ventral attention networks (Fox and Raichle 2007). These networks have been identified with a myriad of techniques, the most common of which, seed-based correlation analysis (SCA) and independent components analysis (ICA), are described below. Furthermore, analysis of complex networks using mathematical approaches (e.g., graph theory) provides insight into local and global properties of specific and whole-brain network organization (Rubinov and Sporns 2010). Such analysis can be useful for identifying differences between populations, such as those at risk for alcoholism and healthy control subjects.

### SCA and ICA

SCA requires a priori selection of a voxel, cluster, or anatomical region, usually based on previous fMRI literature delineating relevant regions of activation or through anatomical delineation. From this a priori selection, time series data are extracted and used as regressors in a linear correlation (or general linear model) analysis from which whole-brain, voxel-wise functional connectivity maps are derived that co-vary with the seed region (for more details see Cole et al. 2010). This approach shows networks of regions that are most strongly functionally connected with the seed voxel or ROI. Conversely, ICA is a data-driven method of analysis that uses whole-brain data to obtain spatially independent and additive components, while assuming statistical independence of non-Gaussian source signals. Networks identified with ICA are compatible with networks found using seed-based methods and typically include less artifactual effects from noise. Additionally, this method can be effective because it eliminates some inherent bias in selecting seed regions (Cole et al. 2010).

Complex network analysis elucidates properties of neural networks beyond simple local correlations established through SCA and ICA. Complex network analysis originated from graph theory but is distinct because it deals with biological networks that are large and complex, like the brain. Nodes and links make up a complex network, which is neither random nor ordered. Nodes typically represent brain regions, whereas links can be represented by anatomical or functional connections. Nodes typically span the entirety of the cortex and do not overlap, whereas links can be unidirectional or bidirectional, or binary, or weighted and represent size, density, or coherence and magnitudes of correlations, or causal interactions in anatomical and functional networks, respectively. The relationships between nodes and edges, in turn, define the network’s topology, which is amenable to descriptive analyses that explore local and global aspects of a network’s organization (Sporns 2014). Node degree, clustering, and modularity are commonly applied measures.

### Structural Connectivity

Neuroanatomic connectivity often is characterized with diffusion tensor imaging (DTI), which provides an indirect measure of white matter^2^ integrity, including myelination and axonal coherence^3^ (Hagmann et al. 2006). DTI assesses diffusion of water molecules in brain tissue. In white matter, water diffusion is restricted and preferentially diffuses along axonal bundles that make up white matter tracts. This restricted diffusion is called anisotropic. Conversely, diffusion of water molecules is isotropic, or less directionally restricted, in other tissues, such as gray matter, indicating more random diffusion. By measuring fractional anisotropy (FA) in the brain, which reflects the degree to which water diffusion is constrained, researchers can draw inferences regarding the underlying white matter microstructure. Because of limitations of DTI in accurately characterizing diffusion in regions with crossing fibers (e.g., regions of prefrontal white matter), researchers must cautiously interpret findings.

## Applications of Neuroconnectivity Analyses to Studies of Risk for Alcoholism

### Family History of Alcoholism and Connectivity

One major risk factor for developing an alcohol use disorder (AUD) is having a family history of alcoholism (Lieb et al. 2002; Schuckit 1985). Neuroimaging research has identified various structural and functional brain differences between youth with familial alcoholism and their peers using volumetric analyses, DTI, and task-based fMRI, which may suggest that there are neural markers of risk, even in the absence of heavy alcohol use (Cservenka and Nagel 2012; Cservenka et al. 2012; Herting et al. 2010; Mackiewicz Seghete et al. 2013; Schweinsburg et al. 2004; Silveri et al. 2011; Spadoni et al. 2013) and in samples with minimal abuse and dependence diagnoses (Hill et al. 2001, 2007, 2011). The available neuroconnectivity tools have been critical for identifying atypical functional connections in at-risk youth, as described below. This avenue of MRI research holds promise for characterizing brain network coherence in studies of familial and genetic risk for alcoholism and is valuable for the examination of connectivity characteristics that could predict future alcohol abuse. Assessing neuroconnectivity in those at risk for alcoholism who have not yet consumed alcohol heavily allows for the distinction between phenotypes related to risk for developing alcoholism and those that could be present as a result of alcohol-induced alterations in brain networks. This advantage may allow future prevention strategies to target their efforts toward risk phenotypes that increase vulnerability for alcoholism, prior to initiation of heavy use.

### Task-Based Connectivity

Task-based functional connectivity has been used in two studies of youth with family history of alcoholism to examine connectivity during working-memory tasks. In a substance-naïve sample of 12- to 14-year-olds, Wetherill and colleagues (2012) examined functional connectivity in working-memory–relevant brain regions, including the bilateral dorsolateral prefrontal cortex (DLPFC) and the posterior parietal cortex (PPC). The BOLD time series were correlated among these seed regions during participant performance of a 6-dot version of the visual working-memory (VWM) task, in which youth had to identify whether dots were the same or different colors after a delay. All fronto-parietal connections examined exhibited weaker synchrony in youth with a family history of alcoholism (FHP) compared with their peers, despite comparable task performance between the groups. Additionally, within the FHP group, there was a significant correlation between number of missed responses and functional connectivity between the PPC and DLPFC. These findings suggest that even in the absence of alcohol or substance use, youth with familial alcoholism already exhibit similar deficits in functional connections in important executive functioning pathways as those seen in alcoholics (Schulte et al. 2012).

Some hypotheses regarding familial risk for alcoholism propose that youth with a family history of AUD may be at greater risk for alcohol abuse as a result of a developmental delay (Corral et al. 2003; Hill et al. 2001). This hypothesis was tested in a functional connectivity study that examined spatial working-memory task connectivity between predefined ROIs in FHP subjects and family history–negative (FHN) youth and compared these functional connectivity patterns to those of an older group of adolescents (Spadoni et al. 2013). Using structural equation modeling, the results showed that the FHP groups differed in connectivity in the right superior parietal lobule to left middle frontal gyrus pathway and that removal of this pathway from the model resulted in a much poorer fit for the FHP group than the FHN youth. These findings suggested that FHP youth differed from their peers in working-memory–related connectivity and that the overall fit of the model for the functional connections among working-memory–related brain regions more closely resembled older adolescents in the FHN sample. These two studies indicate that neural markers for alcoholism may be present during early adolescence when alcohol or substance use has not been initiated and that these patterns may represent a developmental delay in brain network maturity. It will be interesting for future studies to conduct graph theory analyses to examine specific metrics that may differentiate FHP and FHN adolescents in frontoparietal executive functioning systems.

Alcoholics exhibit abnormalities in reward-related structures and atypical reward processing (Makris et al. 2008; Wrase et al. 2007), suggesting that incentive motivational systems may, in part, relate to the risk for alcohol abuse. A study of young adults used the monetary incentive delay (MID) task^2^ to examine functional connectivity of the ventral striatum during incentive versus neutral trials in the MID task (Weiland et al. 2013). FHP young adults showed opposite patterns of connectivity from their peers, such that they exhibited positive functional connectivity between the ventral striatum and sensorimotor cortex, as well as default mode network regions, whereas FHN youth displayed negative functional connectivity between these regions. Additionally, positive functional connectivity between the ventral striatum and supplementary sensorimotor area (SSMA) in FHP youth was positively related to self-reported sensation seeking. A mediation analyses showed that the connectivity between the nucleus accumbens (NAcc) and SSMA mediated the significant association between sensation seeking and alcohol use in the FHP group. It is possible that increased connectivity between reward-related regions and regions involved in motor control could be maladaptive in at-risk youth. The authors proposed that this increased connectivity may represent enhanced and atypical connections between regions involved in reward salience and those important for motor preparation and action. This, in turn, could potentiate actions that involve reward-related behaviors, such as alcohol use.

Fronto-cerebellar abnormalities, including atypical connectivity between the frontal lobes and cerebellum, consistently have been reported in alcoholics (Chanraud et al. 2010; Desmond et al. 2003; Rogers et al. 2012; Sullivan et al. 2003). Because these studies often do not account for preexisting risk factors in adults with AUD, such as family history risk, Herting and colleagues (2011) examined fronto-cerebellar integrity in FHP youth who had no experience with alcohol to examine whether preexisting atypical connectivity of these regions may be a premorbid neural risk feature. Seed-based connectivity of these regions was examined during a variety of fMRI tasks performed by participants in the scanner, that were later averaged. The results from this study suggested weaker fronto-cerebellar connectivity in FHP youth compared with their peers, indicating that previous findings reported in alcoholics may in part be attributed to preexisting risk for alcohol abuse. Additional work is necessary to examine how the integrity of these systems relates to behavioral correlates. This will further increase understanding of the specific deficits that may be associated with weaker integrity of these functional connections, which are likely associated with executive functioning, as such functions have been reported to be mediated by fronto-cerebellar systems (Diamond 2000). Importantly, some of the networks that show FHP-associated alterations in functional connectivity include brain regions where volumetric differences have been identified in FHP individuals, such as the cerebellum (Hill et al. 2007, 2011). This suggests that the underlying basis for altered BOLD synchrony between these regions may be related to premorbid anatomical differences in these structures. Additional work using multi-modal integration of structure and functional connectivity methods is needed to better understand these relationships.

### Resting-State Functional Connectivity

Although more research on familial risk for alcoholism and brain connectivity has focused on functional connections present across task-related BOLD response, recent investigations have examined the intrinsic functional connectivity of brain regions in FHP youth (see figure), specifically using seed-based resting-state connectivity methods. Brain regions and networks that play important roles in reward and emotional processing (e.g., the NAcc and amygdala) often have been the focus of alcoholism research, as task-based neuroimaging studies suggest aberrant brain activity in these areas (Marinkovic et al. 2009; Wrase et al. 2007). Using anatomically defined ROIs of the NAcc on a subject-specific basis, Cservenka and colleagues (2014*a*) found significant differences in the synchrony of both left and right NAcc with other regions of the brain in FHP youth compared with their peers. Specifically, differences were most pronounced in connectivity of the ventral striatum with regions of the frontal lobe. FHP youth had less negative connectivity (or less segregation) between the NAcc and cognitive control regions of the frontal cortex, including bilateral inferior frontal gyri, than their peers. The authors suggested that because reward and executive functioning networks are not as distinctly segregated in FHP youth, this may lead to miscommunication between these regions. Furthermore, this study found that FHP youth had disrupted integration between the NAcc and orbitofrontal cortex (OFC), whereas these regions showed positive connectivity in FHN youth. The authors suggested that reward-related brain areas may be more weakly integrated in FHP youth, which may result in a dissociation between reward response in the brain (mediated by NAcc) and determining the value of rewards (mediated by OFC). Again, it is important to note that alterations in resting state synchrony between the NAcc and OFC may be related to underlying volumetric differences in these regions in FHP individuals. Disruptions in OFC laterality have been previously reported in at-risk youth/young adults (Hill et al. 2009). Associations between functional connectivity and relationships with brain structure require further study.

Recently, another study used RSFC to examine intrinsic connectivity of the amygdala in FHP adolescents and found relationships between behavior in an emotion-cognition task and functional connectivity between the left amygdala and left superior frontal gyrus (SFG) (Cservenka et al. 2014*b*). Weaker connectivity between amygdala and left SFG was associated with poorer impulse control in the context of emotional stimuli in the FHP group. The authors believe that segregation of cognitive and emotional circuitry in at-risk youth may be a marker of weaker cognitive control in FHP adolescents when they are in emotionally laden situations. Because FHN youth displayed mostly positive synchrony between these regions, which was unrelated to task performance, these findings could indicate that once connectivity is established in these regions, it may no longer aberrantly affect behavior. Given discrepancies between children and adults reported in the typical patterns of positive and negative functional connectivity between the amygdala and the frontal lobe (Qin et al. 2012; Roy et al. 2009), more work is needed to determine how the integrity of fronto-limbic circuitry is related to risk for alcoholism. Another finding from this study was an opposite pattern of functional connectivity between the amygdala and cerebellum in FHP youth compared with the pattern observed in their FHN peers (both greater and reduced connectivity, depending on the side of the brain). These results are interesting given previously reported weaker fronto-cerebellar connectivity in FHP youth (Herting et al. 2011), when BOLD signal was averaged across a variety of fMRI tasks. Not only may fronto-cerebellar connectivity be altered in FHP youth prior to heavy alcohol use, but connectivity of these regions with affect-related areas at rest also may be atypical. Interestingly, both reduced contralateral frontocerebellar and amygdalar-cerebellar connectivity was found across both studies (Cservenka et al. 2014*b*; Herting et al. 2011), which supports weaker interhemispheric connectivity between frontal and limbic brain regions with the cerebellum in FHP youth compared with their peers. These findings suggest that both top-down and bottom-up connections with the cerebellum show reduced synchrony across hemispheres in at-risk individuals, a phenotype that merits further exploration, especially given other reports of smaller amygdalar volumes in FHP youth (Hill et al. 2001, 2013*b*).

### DTI

A number of DTI studies have identified white-matter pathways that are altered in high-risk youth and adults (Acheson et al. 2014; Herting et al. 2010; Hill et al. 2013*a*), suggesting that differences in functional connectivity may be related to atypical structural integrity of white matter in FHP individuals. The first study to do so examined white-matter integrity in alcohol-naïve FHP adolescents compared with age- and gender-matched FHN youth and found reduced FA in the superior and inferior longitudinal fasciculi, as well as the anterior superior corona radiata in FHP youth (Herting et al. 2010). Further, reduced FA mediated the relationship between familial alcoholism and reaction times on a delay-discounting task. Because many of these pathways are implicated in connections between brain regions involved in higher-order executive functioning (Seghete et al. 2013; Treit et al. 2013), the findings may reflect either a developmental delay in maturation of white matter, or more lasting deficits in white matter integrity in FHP individuals. Because executive functioning deficits have been observed in both alcoholics (Noel et al. 2007; Verdejo-Garcia et al. 2006) and offspring of alcoholics (Gierski et al. 2013; Harden and Pihl 1995; Nigg et al. 2004), it is plausible that premorbid weaknesses in top-down cognitive functioning and associated neurocircuitry could increase risk for maladaptive decisions regarding alcohol use.

Another DTI study found risk by alcohol exposure effects related to reduced FA in some of the same white-matter pathways previously reported to be altered in FHP youth, including superior and inferior longitudinal fasciculi (Hill et al. 2013*a*). Because this study was conducted in adults, it is possible that developmental timing is a key factor in determining whether risk effects alone are observed and how alcohol exposure may further compromise these vulnerable pathways.

Additional support for lower FA in a variety of white-matter tracts in frontal and parietal regions was recently reported in a large sample of 80 FHP youth, who also had lower FA, compared with their peers in anterior, superior, and posterior corona radiata (Acheson et al. 2014), with the first two pathways exhibiting similar reductions in FA to previously reported findings (Herting et al. 2010). In some cases, studies that have found reduced fronto-parietal functional connectivity in FHP youth have not found reductions in white matter integrity in fronto-parietal pathways (Wetherill et al. 2012). The dissociation between functional and structural connectivity was interpreted as delays in synaptic transmission, rather than compromised myelination of white-matter pathways (Wetherill et al. 2012). However, as a result of the small sample sizes, results need to be replicated.

## Conclusions and Future Directions

As shown, several studies have used neuroconnectivity methods to identify atypical circuitry in the brains of those at familial risk for alcoholism, albeit generally with small sample sizes, which is a limitation of the available research. Overall, these studies have demonstrated abnormalities in connectivity between frontal regions with parietal, ventral striatal, cerebellar, and limbic regions of the brain in these populations, suggesting that these methods may be particularly useful in uncovering neurobiological risk phenotypes. Larger sample sizes and multi-modal method integration are critical to confirm these intriguing findings. Although studies of family history risk for alcoholism have reported atypical functional connectivity using seed-based resting-state and task-based connectivity approaches, none have used graph theory to examine network characteristics of alcoholism-related risk, an analytic strategy which may prove particularly useful for increasing our understanding of the interactions between these networks. Given recent work documenting the amenability of brain functioning to change in response to treatment (Feldstein Ewing et al. 2011), identification of neuroconnectivity treatment targets may substantially increase our capacity to intervene with at-risk populations in a neurobiologically targeted manner.

## Figures and Tables

**Figure:**
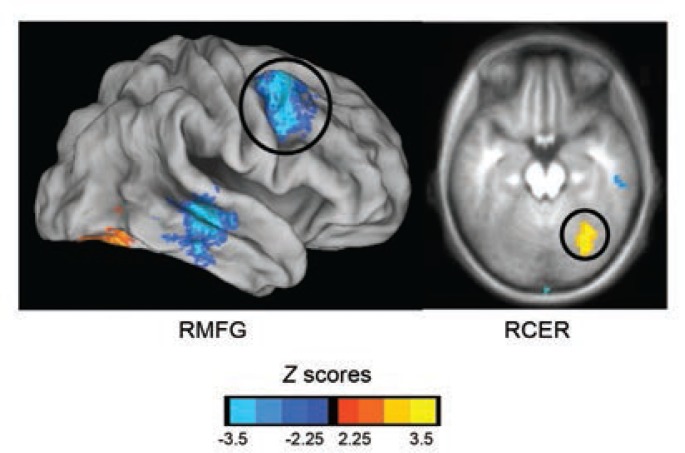
FHP youth have significant differences in right amygdalar resting state functional connectivity patterns compared with FHN youth in frontal and cerebellar regions. This indicates atypical connectivity with executive functioning brain regions in at-risk adolescents compared with controls. RMFG = right middle frontal gyrus; RCER = right cerebellum. SOURCE: Cservenka et al. 2014*b*.

## Footnotes

1 fMRI measures brain activity by detecting changes in blood oxygenation and flow in different areas of the brain.

2 White matter consists of axons surrounded by a protective fatty substance (i.e., myelin) that carry information between brain cells, or neurons, which make up the brain’s gray matter.

3 Axons are the nerve fibers that carry information between neurons.
